# GenAp: a distributed SQL interface for genomic data

**DOI:** 10.1186/s12859-016-0904-1

**Published:** 2016-02-04

**Authors:** Christos Kozanitis, David A. Patterson

**Affiliations:** Department of Computer Science, University of California Berkeley, Soda Hall, Berkeley, 94720 California USA

**Keywords:** NGS, Genomics, Big data, SQL, Interactive, Apache spark, Distributed, Kozanitis, Patterson

## Abstract

**Background:**

The impressively low cost and improved quality of genome sequencing provides to researchers of genetic diseases, such as cancer, a powerful tool to better understand the underlying genetic mechanisms of those diseases and treat them with effective targeted therapies. Thus, a number of projects today sequence the DNA of large patient populations each of which produces at least hundreds of terra-bytes of data. Now the challenge is to provide the produced data on demand to interested parties.

**Results:**

In this paper, we show that the response to this challenge is a modified version of Spark SQL, a distributed SQL execution engine, that handles efficiently joins that use genomic intervals as keys. With this modification, Spark SQL serves such joins more than 50× faster than its existing brute force approach and 8× faster than similar distributed implementations. Thus, Spark SQL can replace existing practices to retrieve genomic data and, as we show, allow users to reduce the number of lines of software code that needs to be developed to query such data by an order of magnitude.

**Electronic supplementary material:**

The online version of this article (doi:10.1186/s12859-016-0904-1) contains supplementary material, which is available to authorized users.

## Background

In this paper, we present an enhancement to Apache Spark, which is a distributed computing framework, to accommodate efficiently SQL queries on genomic datasets. Although one can use existing technologies to import genomic datasets as SQL tables, the poor performance of those technologies to common genomic queries has made them an unattractive choice among users of Next Generation Sequencing (NGS) data.

As sequencing costs drop, more and more research centers invest in massive sequencing projects that aim to build huge databases of thousands of genomes and their associated phenotypic traits. For example the Oregon Health and Sciences University (OHSU) and the Multiple Myeloma Foundation (MMRF) are sequencing 1000 patients with Acute Myeloid Leukemia (AML) and Multiple Myeloma respectively [1, 2]. In another example, the International Cancer Genome Consortium (ICGC) [3] is in the process of sequencing over 25,000 pairs of tumor and normal samples in order to catalogue the genetic abnormalities of 50 different cancer types. Each whole genome sequencing run with Illumina’s technologies produces more than 200 GB of data.

Access to those data, though crucial for the advancement of cancer treatment, remains a challenge for researchers and data scientists.

Today there are two tiers of data access: a top and bottom tier. The top tier involves the downloading of FASTQ, BAM, or VCF files from an archive such as the SRA [4] or CGHUB [5] that contain reads or variants from the sequencing either of a person or a population. Although those archives use the state of the art of file sharing technology to reduce file transfer latencies over the Internet - as is the case of CGHUB that uses GeneTorrent [6] - the size of the files that need to be transfered makes downloading slow. For example, the downloading of a 250 GB BAM with a 60x coverage of Whole Genome Sequencing (WGS) over a 100 Mbps Internet link takes 8 h. On the other hand, the bottom tier involves extractions of subsets of the downloaded data. A user either develops software from scratch to navigate through the data, or they use a combination of shell scripts in combination with commands of either of vcftools, samtools, and BEDtools. This practice adds a layer of complexity between data and the user for three reasons:

1. Scripts must be created to analyze these experiments
2. It requires users to manually parallelize the execution of those tools in distributed environments which get adopted to serve the increasing amounts of generated data.
3. It creates storage overheads with the possible creation of intermediate files that are used to transform files.

Assuming that genomic data in the order of Terabytes and Petabytes reside in distributed environments as in [7], we propose that a more efficient alternative to both tiers of data access is a distributed data retrieval engine. Such an interface on the top access tier can provide data on demand by eliminating the network traffic and the need for secondary level processing at the user end. Even if owners of genomic data repositories are reluctant to provide such a feature, a data retrieval interface is still useful for the productivity of an end user at the bottom layer. With such an interface end users will not have to worry about scripting their way to retrieve and compare data from datasets of different origin, such as raw reads, variants, and annotation data.

In this work we use Spark SQL which is the distributed SQL execution engine of the Apache Spark [8] framework. Spark is a user friendly high performance framework; it abstracts distributed collections of objects and it provides around 80 operators that either transform those objects through opeators such as map, filter, and groupBy, or perform actions on them through operators such as reduce, count, and foreach. Spark organizes a cluster in a Master-Slave architecture, where the driver (i.e., the master) executes a main program and transfers code to workers (i.e. slaves) to execute on those parts of the distributed objects that they contain.

### Data model

In this work we assume that all genomic data are in ADAM format. ADAM [9, 10] is an open source software that separates genomic information from its underlying representation and is currently used as the main computational platform in the NIH BD2K center for big data in translational genomics [7]. Such a separation removes from data users the burden of how data is represented. Thus, an ADAM user can operate on distributed storage without having to parse complicated files, given that ADAM supports and replaces all levels of genomic data that are currently represented by the legacy FASTQ, BAM, and VCF formats.

ADAM records consist of serializable objects that are stored in a distributed friendly column based format. It uses Apache AVRO [11] as a data serialization system, which relies on schemas and stores them together with the data. The serialized data are stored with the use of Apache Parquet [12] system, which is a columnar storage system based on Google Dremel [13]. Parquet creates storage blocks by grouping sequences of records and it stores sequentially all columns of each block. Finally, given that Parquet provides built in support for writes and reads from the Hadoop File System (HDFS), ADAM transparently supports distributed environments that are built over HDFS.

Spark SQL fully recognizes Parquet files and consequently ADAM files as relational tables and it also infers their schemas. This allows users to natively query ADAM files from SPARK SQL.

### The problem

Although Spark SQL provides impressive expressive power and thus it can execute any genomic query, the main obstacle for its adoption to query genomic data has been its slow performance on two of the most frequently encountered queries: 1) random range selection, and 2) joins with interval keys. Random range selection in a collection of aligned reads took several minutes to run in a small cluster, which is embarrassingly slow given that samtools need only a few seconds. Fortunately, the rapid evolution of the open source libraries that we use (in particular Parquet on which ADAM files depend and whose API Spark SQL uses for their filtering) improved the execution of those queries by an order of magnitude as we show in the results section. Regarding the execution of interval joins between two tables, Spark SQL uses the obvious execution of filtering on their cross product. However, given the sizes that are involved in genomic data such an approach is unrealistic. If we consider for example an interval join between 1 billion aligned reads with 1 million variants, the cross product between them is 10^15^ records and it is prohibitively slow to compute.

The contribution of this paper addresses the second performance bottleneck: joins on interval keys. We present a modification to Spark SQL that enhances the efficiency of interval joins and it thus makes it suitable to query genomic data. For this reason we use interval trees to interval join two tables in a distributed setting.

### Related work

The first generation of tools that access genomic data involves packages such as Samtools [14], vcftools [15], BAMtools [16] and BEDtools [17]. While powerful, these tools require extensive programming expertise to open and parse files of different formats, assign buffers, and manipulate various fields. In addition, given that these tools are optimised for single node computational performance, a user needs to manually parallelize them in a distributed environment.

The second generation of relevant software involves the Genome Query Language (GQL) [18], which provides a clean abstraction of genomic data collection through a SQL-like interface. However, the support of GQL is only limited to queries on a small subset of fields of the entire SAM specification and it also requires extra manual effort to support distributed environments.

The third generation leverages the Hadoop ecosystem to easily provide data on demand on a distributed setting. For example, the GenomeMetric Query Language (GMQL) [19] uses Apache Pig, which is a high level language that abstracts map-reduce operations, to support metadata management and queries between variants and annotation data. In another example, NEXTBIO [20] uses HBase, which is Hadoop’s NoSQL key-value store, to support data of similar nature. The scope of these tools however excludes raw data either in the FASTQ or in the BAM format.

## Implementation

This section describes how we modified Spark SQL to add support for range based joins. The first step of the modification involves the grammar of Spark SQL, which we extended to simplify the syntax of those queries. Next, before describing our modification to the execution engine of Spark SQL, we provide a brief description of the interval tree and interval forest data structures which this modification utilizes.

### Syntax

Although the existing syntax of Spark SQL suffices for a user to describe a join between two tables on interval overlap condition, it looks complex and counter intuitive for a user of genomic collections that routinely uses this operation. If we consider for example tables A (aStart: long, aEnd: long, aChr: String) and B (bStart: long, bEnd: long, bChr: String), then according to the current syntax of Spark SQL, an interval join looks like the following:



To eliminate the complexity of such a frequent operation, we enhanced the vocabulary of Spark SQL with two extra keywords, namely *RANGEJOIN* and *GENOMEOVERLAP*. In case of an interval based join, the former keyword replaces *JOIN* and the latter, which is followed by two tuples as arguments, specifies the overlap condition and is the only argument of the *ON* condition.

Using those new keywords, one can type the query of the previous example as follows:



### Interval trees

The most expensive part of a Join evaluation involves a search for overlaps between two arrays of intervals. Our implementation utilizes the interval tree data structure, which is a binary tree that is built from a collection of *n* intervals in O ($n \log n$) time and it takes O ($\log n$) time to find which of the intervals of its collection overlap with a given query interval. Note that a brute force execution, which is what Spark SQL uses up to date, of the same operation takes quadratic time.

At this point we remind interested readers how an interval tree is constructed and searched.

**Fig. 1:**
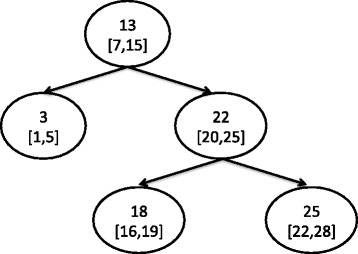
An example interval tree. This is the interval tree that stores intervals [1,5], [7,15], [16,19], [20,25], and [22,28]. Each node consists of two parts: The top part is the key of the node, which is the midpoint of the concatenation of all intervals of the subtree and the bottom part is a list of intervals that overlap with the key. So, in this example, the concatenation of all intervals is [1,28] and since the midpoint of that is 13, the root of the tree is keyed by 13 it stores interval [7,15] as it is the only interval of the collection that overlaps with 13. The intervals that do not overlap with the midpoint are used to build the left and right subtrees recursively such that all intervals of the left subtree end before and all intervals of the right subtree start after the midpoint

Each node of the tree contains two objects. The first is the *key* that is the mid point of the union of the collection of intervals that are stored in the subtree which is rooted at a node. The second object is an overlapping list that contains those intervals that overlap with the *key*. Consider for example the interval tree of Fig. 1 which stores intervals [1,5], [7,15], [16,19], [20,25] and [22,28]. The *key* of the root is 13 because that is the mid point of the union of all intervals which is [1,28]. This key overlaps only with interval [7,15], which is the only content of the overlapping list of the root.

The subtrees of a node store those intervals that do not overlap with its *key*. The left subtree contains all intervals the end points of which are less than the *key*; symmetrically, the right subtree contains all intervals with start points greater than the *key*.

To search whether a particular interval overlaps with any of the intervals of an interval tree, one scans linearly the overlapping list of the root to search for intervals that might overlap with the query and continues traversing the tree according to the relevant position of the query interval and the *keys* of the encountered nodes. When the input interval ends before the *key* of a node the search continues only to the left subtree. Respectively, when the start of the query interval is greater than the *key* the search continues only to the right. In case of overlapping between an input interval and a *key*, the search continues to both subtrees. Assume for example a search for all overlapping intervals with [17,23] with the interval tree of Fig. 1. Starting from the root, after a quick scan of the overlapping list of the root returns the empty set, a comparison between the *key* of the root and the interval indicates that the search should continue towards the right subtree, which is rooted at the node with 22 as *key*. A quick scan of the overlapping list of the node detects that [20,25] is part of the solution and since the query interval overlaps with the *key* of the node, the search continues towards both left and right subtrees. Proceeding in the same way, the query returns as a result the set of intervals [20,25], [16,19] and [22,28].

### Interval forests

The interval tree structure that we described in the previous session is not useful as is to query genomic intervals because it does not differentiate intervals of different chromosomes. Thus, in this work we use a forest of interval trees, one per chromosome. All chromosome names are stored in a hash table and they point to the respective interval tree. Thus, a genomic interval lookup now consists of a hash table lookup to obtain the proper interval tree for the chromosome of interest before querying the tree as the previous subsection describes.

### Interval keyed joins on Spark SQL

**Fig. 2:**
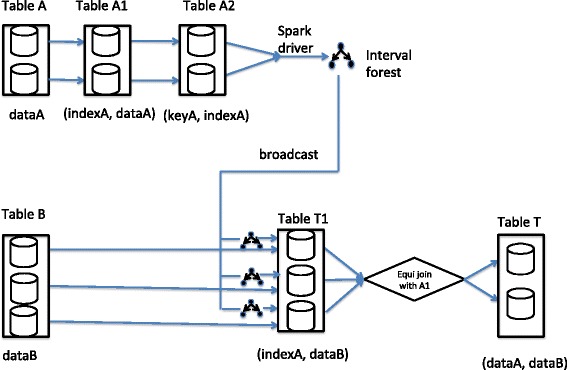
Our distributed range join architecture. In this picture, distributed table A joins with distributed table B on genomic interval overlapping. Table A goes through a number of transformations to enable the Spark driver to create an interval forest which stores index pointers to the original data. Next it propagates the interval forest to workers which transform table B by performing interval lookups on the forest. The result of this operation is table T1, which contains tuples of data from table B and pointers to data of table A. To materialize this we join it with table A1 on those pointers and we obtain table T, which is the final result of the operation. The text under each table shows the data type of the contents of each table

Figure 2 shows the implementation of the join between distributed tables A and B with interval keys.

In the first stage of the join between distributed tables A and B on interval keys and assuming A is the table with the fewest records, Spark SQL first creates distributed table A1 which is a transformation of table A into a key-value pair whose keys are the indices of each record. Note those indices are easy to obtain in Spark using the built-in function *zipKeysWithIndex*. Then it creates table A2 which is a key-value pair where the keys are the intervals of interest and the values are the keys of table A1. For example, consider table A whose schema is 〈*id*(*string*):*chromosome*(*string*):*txStart*(*long*):*txEnd*(*long*):*function*(*string*)〉. If table A contains entries 〈 “ *gene*1" :*chr*3:100:1000: “ *function*1" 〉 and 〈 “ *gene*2" :*chr*3:4000:5000: “ *function*2" 〉 then table A1 is

$$\begin{array}{*{20}l} \langle 0 &: ({}^{\prime\prime}gene1^{\prime\prime}, chr3, 100, 1000, {}^{\prime\prime}function1^{\prime\prime}) \rangle \\ \langle 1 &: ({}^{\prime\prime}gene2^{\prime\prime}, chr3, 4000, 5000, {}^{\prime\prime}function2^{\prime\prime}) \rangle \end{array} $$

and the contents of table A2 are

$$\begin{array}{*{20}l} \langle (chr3, 100, 1000):0 \rangle \\ \langle(chr3, 4000, 5000): 1\rangle \end{array} $$

In the second stage of the implementation, the driver of the Spark cluster collects A2 into its local memory and it uses it to create an interval forest according to the description of the previous subsections. Then the driver broadcasts the resulted interval forest to the worker nodes.

In the third stage, Spark SQL creates table T1 with records that consist of a tuple whose first element involves a record from table B and its second element is one of the *index* values of table A2. In order to create table T1, Spark SQL transforms table B by using its interval keys to look up the interval forest that all worker nodes obtained from the previous step. For example consider table B with schema 〈*id*(*string*):*chromosome*(*string*):*readStart*(*long*):*readEnd*(*long*)〉. If table B contains entries

$$\begin{array}{*{20}l} \langle {}^{\prime\prime}read1^{\prime\prime} &: chr3 : 150 : 250 \rangle \\ \langle {}^{\prime\prime}read2^{\prime\prime} &: chr4: 3000: 3100 \rangle \\ \langle {}^{\prime\prime}read3^{\prime\prime} &: chr1 : 1000 : 1100\rangle \end{array} $$

and A2 is the table that we created in the previous paragraph whose interval keys created the interval forest, then only the first tuple of table B will query successfully the interval forest since its interval overlaps with the first interval of table A2. Thus the contents of table T1 are $\left \{ \langle ({}^{\prime \prime }read1^{\prime \prime }, chr3, 150, 250) : 0 \rangle \right \}$.

The last stage of the operation involves the generation of the final distributed table, which is the result of the range join and it consists of a tuple where the first element involves a record from table A and the second element involves a record from table B. In order to construct the output, Spark SQL joins table T1 with table A1 on the integer keys of A1 which have to match with the second element of the tuples of T1. So, in our example the transformation of T1 gives table T with contents $\left \{\langle ({}^{\prime \prime }read1^{\prime \prime }, chr3, 150, 250):({}^{\prime \prime }gene1^{\prime \prime }, chr3, 100\right.$, $\left. 1000, {}^{\prime \prime }function1^{\prime \prime })\rangle \right \}$

## Results

We demonstrate the suitability of Spark SQL on querying genomic datasets through a series of queries that involve random access of ranges and range joins. In our examples we used the ADAM representation of the raw data (BAM file of size 250 GB) of the platinum genome NA12878. Our hardware consisted of a Spark cluster with 5 workers all of which were m2.4xlarge instances on the Amazon EC2cloud.

We start by demonstrating the maturation of the technologies that we use through a random access of a simple range of gene NPM1. The query that we type is the following:



We ran the query against two different versions of the Parquet library that comprises the storage model of ADAM and whose API Spark SQL uses to evaluate filter predicates on Parquet encoded files. The earlier version of this library took 10 min to run while the latest version took 75 s. The difference was due to the fact that although the earlier versions of Parquet were evaluating a predicate on every single record of a file, recent ones utilize metadata to ignore retrieving unnecessary blocks of data. Of course, a minute long latency for such a query still seems incompetent compared to the second long latency that samtools obtain in single node through secondary indexes, but the observed trends make us believe that future improvements on the used libraries are going to bridge that gap.

In the second query, we demonstrate the efficiency of our implementation of interval joins. Note that this query joins all mapped reads of NA12878 which is in the order of 10^9^ records with all 94 K records of the clinvar vcf that is provided at the dbsnp archive for build 142. Of course, the brute force solution which is a filtering operation on the cross product of the two tables is unrealistic since the cross product consists of a number of records in the order of 10^14^. The query that we used is:



Table 1 compares the performance of our implementation with the brute force original implementation of Spark SQL and the shuffle join approach that is introduced in [10] as an alternative range join operation over genomic sequences. According to this table, the interval tree based method that we use in this paper is 9 times faster than shuffle join while the brute force approach could not complete the join by the time we interrupted its execution.

**Table 1:** Performance of different implementations of interval keyed joins

Method	Runtime (hr)
Interval Tree (this paper)	0.28
Shuffle Join (ADAM)	2.5
Brute force (default Spark SQL)	>14

**Fig. 3:**
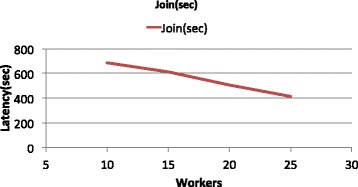
Scalability of range join. The execution time drops linearly as the cluster size increases

The next experiment demonstrates the scalability of our range join implementation by modifying the number of Spark workers that are used for the execution of the previous query. Figure 3 shows that the latency of the interval tree based join drops linearly as the size of the cluster increases.

To examine the small slope of the curve of Fig. 3, which indicates small performance gains due to the cluster size increase, we monitored closely the utilization of the clusters. We focused mostly on the drop of the CPU utilization at points 3:26 and 3:30 of Additional file 2: Figure S1 A. In the absence of any memory bottlenecks or intense network activity (Additional file 2: Figure S1 B and C respectively), such a drop in CPU utilization indicates the existence of stranglers, i.e. a small subset of workers that still execute their part of a task while most nodes wait for them to finish in order to get assigned a new task. Although a complete understanding of strangler behavior in distributed systems is still an open research problem [29], the nature of the dataset, which is sorted per chromosome location, makes us estimate that most probably the existence of stranglers occurs due to undesired, in this case, data locality. Given that the Hadoop File System partitions data blindly and our original input was initially sorted, it is very likely that overlapping data are grouped in the same partition and thus they are assigned to the same worker. So, when Spark SQL attempts to perform r ange join, it is likely that not all workers contribute equally to the preparation of the output; those whose most of the data do not participate in the join are going to complete their tasks earlier than workers that produce most of the output. We leave as a future work research on the best possible partitioning of genomic datasets in order to get queried as efficiently as possible.

Our final experiment stresses the tremendous savings in programmer productivity that the use of Spark SQL yields compared to the alternatives that researchers use today. The query that we used in this example was: “Are there any reads of the NA12878 dataset that align with the reference genome only with matches or mismatches (M CIGAR operations only), overlap with any variant location of the clinvar dataset and contain the respective alternate allele?”. Using existing genomic processing software, we implemented the same query in two steps:

1. We used the *intersect* feature of BEDtools to retrieve all those alignments of NA12878 that overlap with any locus of the clinvar dataset.
2. We wrote custom software using the samtools API to further process the retrieved alignments and discard those whose reads contain the reference allele.

The custom software that we wrote and we also include as an additional file (see: Additional file 7) consists of 130 lines of C++ code. The execution of both steps took 27 min to run; BEDtools took 26 min to filter the dataset and the resulted file was processed by our custom software in less than a minute. On the other hand, the Spark SQL implementation of this query requires an interval based join between the contents of NA12878 and clinvar tables followed by a *WHERE* statement that filters out rows with either complex CIGAR strings or with sequences that do not contain the alternate allele in the respective positions; the resulted Spark SQL script, which we also include as an additional file (see Additional file 6; interested readers can experiment with the code of this file against small datasets that we provide in Additional files 3, 4 and 5), contains 11 lines of code that took 16 min to run on the same hardware.

Table 2 summarizes the results of this experiment. They show a decrease by an order of magnitude on the number of lines of code that needs to be developed with Spark SQL to implement complex queries on genomic data. In addition, the execution of the Spark SQL query was faster than the execution of BEDtools. The reason was that the column based storage and predicate push-down capabilities of the ADAM format that we used to represent alignments allowed Spark SQL to retrieve from the disk only the absolutely necessary fields of the dataset.

**Table 2:** Comparison between Spark SQL and existing software methods that are used to retrieve genomic data. Complex queries that today need more than a hundred lines of code to be implemented, take an order of magnitude fewer lines of code on Spark SQL without performance sacrifices

Software tool	Lines of code	Runtime (min)
Spark SQL	11	16
State of the art software	BEDtools: 1	26
	samtols API based code: 130	1
	total: 131	27

## Discussion

In our work we utilize the latest technologies in distributed computing, such as Spark SQL through Apache Spark and Parquet through ADAM in order to enable the research community to easily query heterogeneous datasets of genomic data either against each other (for example mixed queries between variant and raw data) or with annotated files.

Out of the two categories of software that query large amounts of data, NoSQL systems and distributed SQL execution engines, in this work we use a distributed SQL execution engine to address these needs because of its expressive power, and its scalability. As traditional relational databases are hard to scale, large amounts of data can be accessed via NoSQL systems such as MongoDB [21], HBase [22], and Cassandra [23]. However, in the light of [24] which advocates the use of relational algebra to retrieve genomic data, access patterns to sequencing data typically involve a combination of operators such as projections, selections, interval joins, and aggregations; NoSQL systems do not support all these operations. On the other hand, distributed SQL execution engines, such as as Hive [25], Impala nature [26], and Spark SQL [27] provide scalable access to the data through relational algebra based interfaces.

Of course, Spark SQL is not the only distributed SQL execution engine, but we chose it in this work because of its sophisticated and extensible query planner. Big Data users have been using distributed SQL execution engines as an abstraction of distributed operations, such as map and reduce, through relational algebra operations. Earlier engines, such as Shark [28], Hive [25], and Impala [26] rely on a step-by-step execution of the SQL statements. Spark SQL, on the other hand, provides a modular query planner and an optimizer to increase the performance of the execution. In this work, we use the modularity of the query planner to enhance the execution of some common queries.

## Conclusions

The central message of this paper is that a state of the art distributed SQL execution engine, such as Spark SQL, can be modified to provide an interactive SQL interface on all kinds of genomic data. Of course, other tools, such as samtools and bedtools can provide similar functionality, but Spark SQL provides a simple and expressive mechanism of querying heterogeneous genomic data over distributed hardware setups. And we expect that as big data technologies mature, the performance of Spark SQL is going to match the performance of today’s popular tools.

## Availability and requirements

- **Project name:** GenAp
- **Project homepage:** branch rangeJoinsExtraPredicates-1.3 of https://github.com/kozanitis/spark. see Additional file 1 for instructions on how to clone and build the project.
- **Operating systems:** Linux
- **Programming Language:** Scala
- **Other requirements:** Maven, Java. We verified that the project builds with Maven 10.10.4 and runs with Java version 1.7.0_51
- **Licence:** Apache 2

## Additional files

Additional file 1
**This file contains instructions on how to clone and build our modification of Apache spark and run it in local mode on a user’s computer.** (TXT 1 kb)

Additional file 2
**This is a file that contains the supplementary figures that we refer to in the manuscript.** (PDF 615 kb)

Additional file 3
**This tarball contains a sample file of aligned reads in ADAM format.** Note that this is a binary format and we provide a user the oportunity to explore this file by attaching the equivalent SAM file of the same alignments. (TAR 21 kb)

Additional file 4
**The SAM equivalent of the Additional File** 3. (SAM 3 kb)

Additional file 5
**This is a sample vcf file similar to the one we used in the results section.** (VCF 9 kb)

Additional file 6
**This is sample code that uses Spark SQL to perform an interval based join to the contents of sampleReads.adam with the contents of Additional File** 5. (SCALA 1 kb)

Additional file 7
**This tarball contains the custom C++ software that we wrote to implement the last query of the results section.** For reference, it also contains the respective Spark SQL implementation. (TAR 9 kb)

## Abbreviations

NGS: next generation sequencing
OHSU: oregon health and sciences university
MMRF: multiple myeloma foundation
AML: acute myeloid leukemia
ICGC: international cancer genome symposium
WGS: whole genome sequencing
HDFS: hadoop file system
GQL: genome query language
GMQL: genomeMetric query language
