# Supplementary material for: GenAp: a distributed SQL interface for genomic data
Source: BMC Bioinformatics. 2016 Feb 4;17:63. doi: 10.1186/s12859-016-0904-1 (PMC4741060; doi:10.1186/s12859-016-0904-1)
Supplement: Additional file 2 — This is a file that contains the supplementary figures that we refer to in the manuscript. (PDF 615 kb) [file 12859_2016_904_MOESM2_ESM.pdf]

Supplementary Matterial

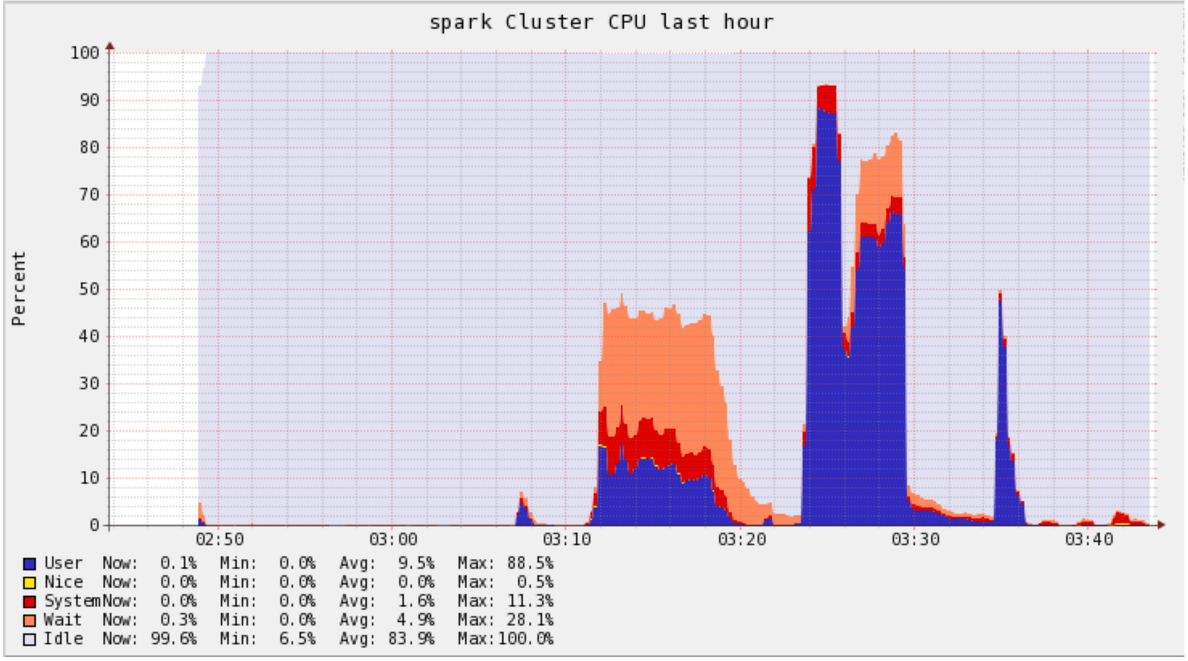

(A)

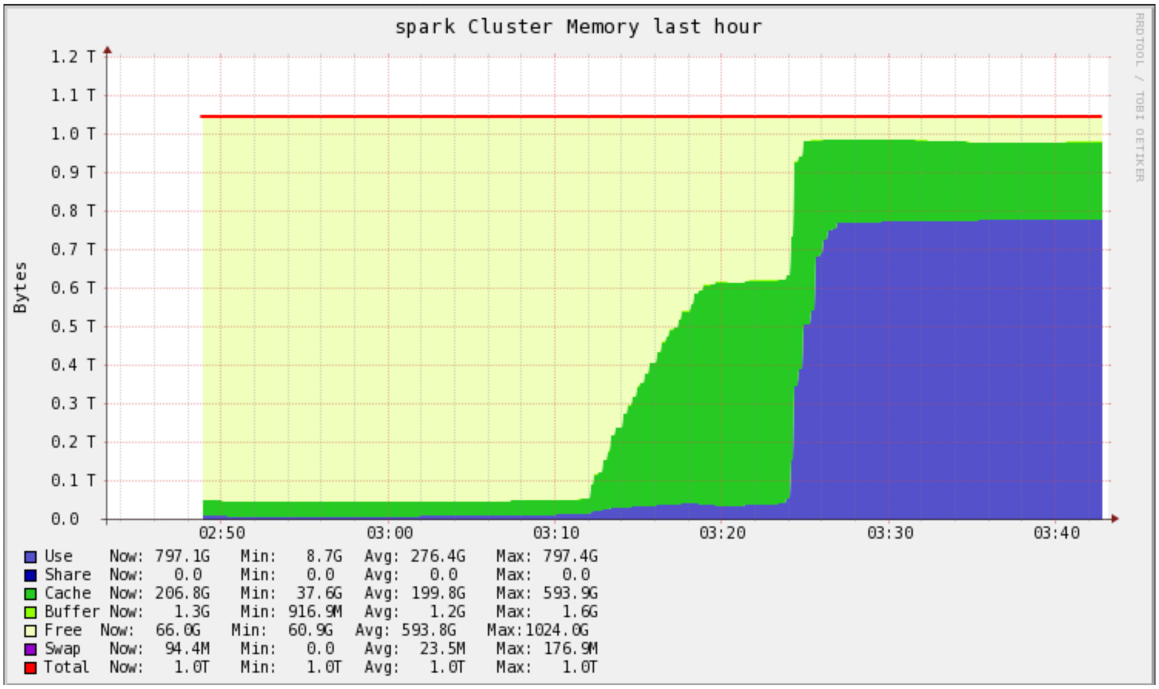

(B)

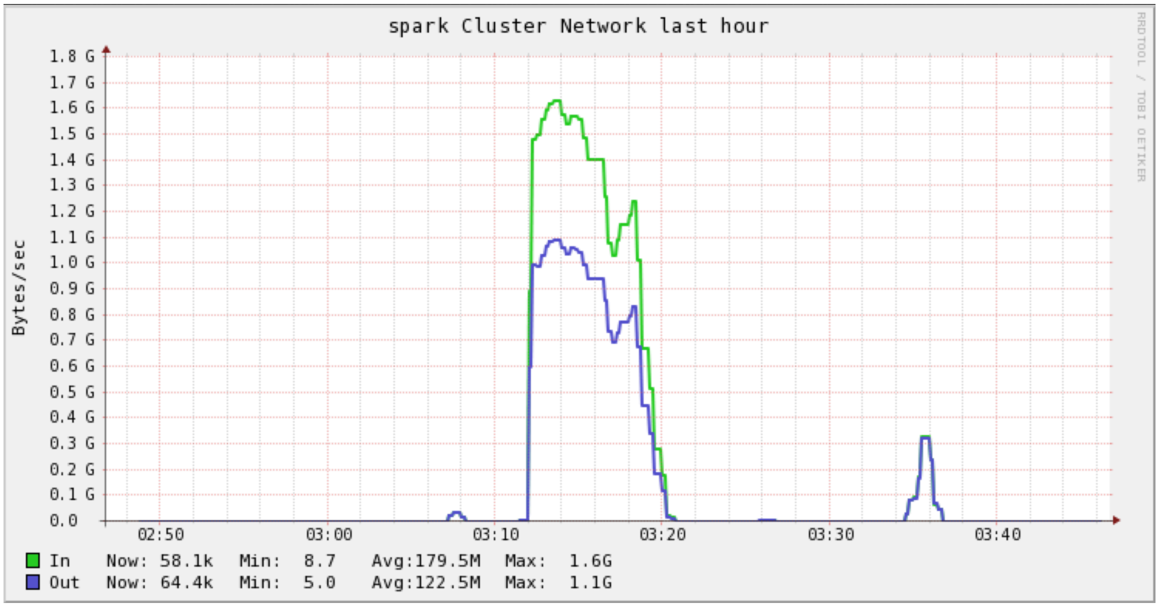

(C)

Supplementary Figure 1. Utilization statistics of the "scale" experiment execution on a 15-worker cluster. The figures are screenshots from the ganglia monitoring system. Note that the experiment that we describe in the main text of the paper started at time 03:24.
